# Improving equity, diversity, and inclusion in academia

**DOI:** 10.1186/s41073-022-00123-z

**Published:** 2022-07-04

**Authors:** Omar Dewidar, Nour Elmestekawy, Vivian Welch

**Affiliations:** 1 Bruyere Research Institute, University of Ottawa, Ottawa, ON Canada; 2 Faculty of Health Sciences, University of Ottawa, Ottawa, ON Canada; 3 School of Epidemiology and Public Health, University of Ottawa, Ottawa, ON Canada

**Keywords:** Equity, Diversity, Inclusion, Research Integrity, Journal policies, Editorial bias

## Abstract

There are growing bodies of evidence demonstrating the benefits of equity, diversity, and inclusion (EDI) on academic and organizational excellence. In turn, some editors have stated their desire to improve the EDI of their journals and of the wider scientific community. The Royal Society of Chemistry established a minimum set of requirements aimed at improving EDI in scholarly publishing. Additionally, several resources were reported to have the potential to improve EDI, but their effectiveness and feasibility are yet to be determined. In this commentary we suggest six approaches, based on the Royal Society of Chemistry set of requirements, that journals could implement to improve EDI. They are: (1) adopt a journal EDI statement with clear, actionable steps to achieve it; (2) promote the use of inclusive and bias-free language; (3) appoint a journal’s EDI director or lead; (4) establish a EDI mentoring approach; (5) monitor adherence to EDI principles; and (6) publish reports on EDI actions and achievements. We also provide examples of journals that have implemented some of these strategies, and discuss the roles of peer reviewers, authors, researchers, academic institutes, and funders in improving EDI.

## Background

Editors, reviewers, researchers, funders, and academic institutions collectively act as gatekeepers of our disciplines. Their unique positions enable ethical publication practices and the setting of rigorous research standards. Frequently, these stakeholders are tasked with making critical judgements that can help progress our fields. In some cases, these judgements may be unintentionally biased and possibly fueled by the spread of misinformation.

The academic publication process is built on objectivity [1], gender and socio-cultural neutrality [2], and respect for human and animal rights. Hence, equity, diversity, and inclusion (EDI) are essential in publication processes, among other academic spaces. However for the purpose of this According to the Editors Association of Canada [3], equity refers to recognizing the existence of “identity-based advantages and barriers” as well as “working to correct and address this imbalance.” They also define diversity as “increasing the presence of people of diverse identities” in the editorial process and inclusion as “creating an environment where all those with diverse identities are welcomed and valued”.

Given the ‘publish or perish’ nature of academia, the role of Journals and editors in propagating the cycle of injustice in this space is amplified [4]. There is evidence for a higher rejection rate of papers from traditionally under-represented groups [4, 5]. These decisions can heavily impact such individuals, resulting in poorer career progression due to fewer publications and a lower chance of promotional opportunities. The obstruction of career progression contributes to the lack of representation of certain groups in positions of power and leadership: particularly women, individuals living in low-middle income countries and racialized people [6–12]. For example, in oncology research, Caucasian men hold over 70% of editorial leadership positions [6]. Similar findings were shown in a survey of editors of the Association of College & Research Libraries [7], and Wiley publishing [13]. Furthermore, in communication journals, editorial board members from the United States are more than all other world regions pooled together [14]. It has been hypothesized that overrepresented groups may have implicit biases that stem from historical institutionalized discrimination against individuals from under-represented groups [15, 16]. However, the evidence is conflicting. Witteman and colleagues [17] demonstrated that when controlling for age and domain of research, a gender bias exists in peer review processes that are judging the calibre of the investigator: there is a 4% lower success rate for women. Yet, a more recent large analysis of 145 journals found that the bias is non-existent [18]. In fact, women led, and co-authored articles were favoured by referees and editors [18]. Nonetheless, some studies have demonstrated that implicit bias training may lead to modifying behavior [19–22]. Thus, EDI training and other resources, such as unconscious bias [23, 24] and indigenous cultural competency training [25, 26], should be easily accessible and completed by the editorial teams and authors alike.

Realizing that biases exist in scholarly publishing, The Royal Society of Chemistry (RSC) established a joint commitment to action on EDI in scholarly publishing. In collaboration with signed partners, they formulated the following six minimum standards for inclusion and diversity in academic publishing: (1) integrate inclusion and diversity in the publishing activities and strategic planning; (2) work on understanding the demographic diversity of individuals at all levels of their publishing process; (3) acknowledge and address the barriers experienced by those who are under-represented among them; (4) define and clearly communicate diversity and inclusion responsibilities at all levels of the publishing process; (5) revise the appointment process for editors and editorial boards as needed, to widen the scope of the captured talent; (6) publicly report diversity and inclusion progress at least once a year [27]. To date, 52 publishing organizations have committed to this initiative [27].

It may be argued that editors should not be obliged to ensure that their reviewer pool is geographically distributed, and that their only concern should be recruiting reviewers who are experts of the manuscript content under consideration. However, the lack of diversity in the peer reviewer can make finding reviewers harder [28]. In addition, there are many benefits to promoting diversity in the publishing processes for the scientific community. Ensuring the representation of individuals from underrepresented populations could facilitate meaningful career growth for these individuals and increase the depth of the content published in the journal. An environment of innovation and creativity could be fostered through the presence of a greater variety of problem-solving approaches [4, 29]. Better performance, predictions, and overall results could emerge as problem-solving improves in the presence of a diverse team [30]. It was found that a significant increase in the citation of articles occurred when the authors who wrote them were of different ethnicities and nationalities [30]. Additionally, there was an association between the 5-year citation count of published papers and the diversity of people who authored them — ethnic diversity in particular [31]. For example, when a mandate was instituted in Japan by the Okinawa Institute of Science and Technology Graduate University to ensure 50% of all researchers were from other than Japan, the institute saw an increase in academic ranking based on their research output [32].

**Fig. 1:**
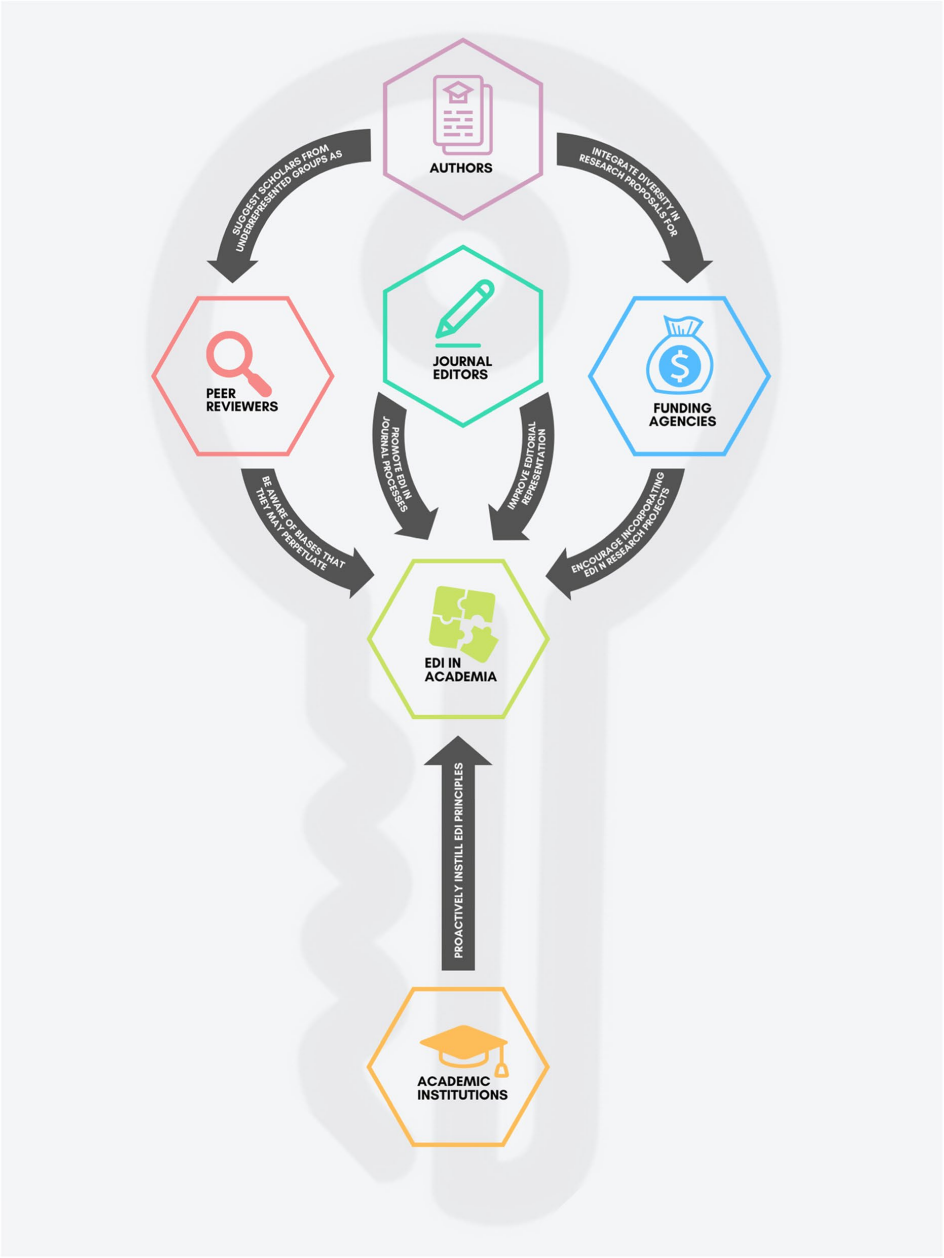
Key model for improving equity, diversity, and inclusion (EDI) of journals. This figure was generated by the authors using Canva (https://www.canva.com/)

Although commitments are in place to improve EDI in journals and publishing [33–37], the effectiveness of these approaches are yet to be determined. We also acknowledge that editor of this journal shared concerns for practical approaches to improving EDI in peer review and journal practices [38]. In this commentary, we provide practical approaches for editors and journal publishers to improve EDI in academic journals based on the six minimum standards set by the RSC. In Table 1 we also provide examples of journals that implemented some of these strategies. Finally, EDI issues in academia are tightly intertwined with systemic oppression that is integrated in policies and regulations of academic progression. Thus, both a bottom-up and top-down approach are needed to induce change. Subsequently, we reflect on the role of reviewers, researchers, academic institutions, and funding agencies in shaping the academic ecosystem. Figure 1 presents how these stakeholders contribute to fostering a more equitable, diverse, and inclusive academic community.

**Table 1:** Recommendations for improving equity, diversity, and inclusion (EDI) journals

**Recommendations**	**RSC minimum standard**	**Example**
**Adopt a journal diversity statement with clear, actionable steps to achieve it**	*Acknowledge the barriers within publishing which authors, editorial decision makers and reviewers from under-represented communities experience and take actions to address them.*	“BMJ aims to support clinicians and researchers from all over the world to have good quality work published, regardless of their sex/gender, race/ethnicity, first language, sexual orientation, religion/beliefs, disability status, age/status, or nationality/citizenship. We are trying to help dismantle the barriers that have previously prevented underrepresented groups from seeing their work published and therefore help them to advance their careers.” [39]
**Promote the use of inclusive and bias-free language**	*Ensure inclusion and diversity are integrated into publishing activities and strategic planning.*	“We have reviewed internal initiatives, practices, and policies across the journal family that promote diversity and inclusion in our editorial content and within our teams. For example, we have updated our language and style guide to ensure consistent use of inclusive language.” [40]
**Appoint a journal’s EDI director or lead**	*Define and communicate the specific responsibilities authors, editorial decision makers, reviewers and staff members have towards inclusion and diversity.*	ElSEVIER have an Inclusion & Diversity Advisory Board aimed at driving initiatives for improving gender, race & ethnicity diversity in academic research and assist in setting standards for unbiased robust decisions. The members vary in gender, specializations with some members experienced in leading diversity working groups in academic spaces. [41]
**Establish an EDI mentoring approach**	*Review and revise as appropriate the appointment process for editors and editorial boards to capture the widest pool of talent possible.*	“*Journal of Applied Ecology* runs a mentoring opportunity for early career researchers who are interested in learning about the Associate Editor role and have little or no previous editorial experience. Participants work with the Senior Editors who provide guidance on handling papers during a two-year Associate Editor training post.” [42]
**Monitor adherence to EDI principles**	*Work to understand the demographic diversity of authors, editorial decision makers and reviewers, such as gender, geographical location, and ethnicity data.*	“The Workplace Equity Project (WE) conducted a global survey to capture data about workplace experiences, practices, and opportunities in the scholarly publishing industry. The WE survey was open to everyone who works in this sector as publishers, service providers, and across the spectrum of related organizations, companies, and consultancies.” [43] “The survey was crowd-sourced and reviewed by a diverse team of industry professionals to ensure that the questions are oriented to a global audience, cover relevant areas, and do not contain inherent bias. To encourage widespread participation in the survey, we have secured endorsements from major industry organizations. The Society for Scholarly Publishing (SSP), International Association of Scientific, Technical, and Medical Publishers (STM), National Federation of Advanced Information Systems (NFAIS), and UN Women UK will assist in its promotion and distribution to their members and followers.” [43]
**Publish reports on EDI**	*Publicly report on progress on inclusion and diversity in scholarly publishing at least once a year.*	“In this annual report we are pleased to provide information on gender, race/ethnicity, and region of the world of authors and gender of peer reviewers. Findings allow the editor team to quantify under-representation and identify bias in peer review that might be impairing progress in science, and adversely affecting thrombosis and hemostasis research and representation among the science community.” [44]

*RSC* Royal Society of Chemistry (https://www.rsc.org/new-perspectives/talent/minimum-standards-for-inclusion-and-diversity-for-scholarly-publishing/), *EDI* Equity, diversity and inclusion

## Guidance

### The role of journals

Given that the impact of journal policies on compliance to mandates has been demonstrated in several areas, such as clinical trial registration and reporting guidelines [45–47], editors and publishers should articulate a framework that influences the incorporation of EDI. We propose below six approaches that align with the six RSC recommendations for improving EDI in academic publishing.

1. ***Adopt a journal diversity statement with clear, actionable steps to achieve it***
  Increasing diversity and inclusion in scientific publishing enhances excellence and innovation. Adopting a journal diversity statement, with clear, actionable steps to achieve it, is a practical first step for defining the problem and establishing accountability [37]. Explicitly defining the problem helps ensure that everyone shares the same understanding of it. Moreover, this process engages senior leadership to support EDI principles, making it clear to authors, reviewers, and editors that change is a priority. Several reports show that these recognition schemes provide an impetus for action on EDI which translates to more inclusive environments [48]. More than 47 publishing organizations have adopted recognition schemes [49]. Wiley publishing has developed guidance for assisting editors in developing an EDI statement [50]. The process involves the following three steps: (1) assessing the journal and research community’s needs, (2) identifying action priorities for the journal (I.e., changes in recruitment process, improving the diversity of invited reviewers), and (3) developing an active statement that acknowledges that this process is an ongoing one that will require revisiting on a regular basis to answer unknown questions.
2. ***Promote the use of inclusive and bias-free language***
  Avoiding the perpetuation of prejudicial beliefs or demeaning attitudes in publishing activities may improve the recruitment of populations experiencing disadvantages. In turn, the journal should promote the use of inclusive and bias-free language in all correspondence and the journal website content [51]. With changes in language over time, editors should address individuals and or communities as they prefer to describe themselves, their experiences, and practices. For example, a notable addition to the 7^th^ edition of the America Psychological Association is the recommendation to use the singular “they” to refer to individuals when the identified pronouns are unknown or hypothetical person is irrelevant within the context [37]. The University of Nottingham reported improvements in the recruitment of female researchers in science, technology, engineering, and mathematics (STEM) when the language of advertised fellowships was checked for gender inclusivity among schemes [48, 52, 53].
3. ***Appoint a journal’s equity, diversity and inclusion director or lead***
  When leaders use the power associated with their positions to advocate for EDI this may help support others to eradicate prejudice and discrimination. Editors in chief should prefer to include scholars with underrepresented backgrounds and EDI expertise to lead in EDI advocacy roles. They could, albeit less preferable, nominate one of their associate editors who has an underrepresented background or recruit an individual with expertise on EDI who does not have an underrepresented background. It would be wise to create a consultation committee for the EDI lead composed of underrepresented academics, EDI leaders, and members of the public with unique, lived experiences. The perspectives of underrepresented individuals could be crucial for the team’s success as it would help produce more culturally competent and practical solutions. The responsibilities of the lead could include reviewing journal processes while working with the Editors in chief, raising awareness of unconscious bias among the editorial teams and implementing initiatives that could improve EDI. The lead should also be responsible for developing strategies that would diversify the editorial teams, peer reviewers and authorship as well as monitor the journal’s progress in achieving EDI. The individual or team leading this appointment should review the journal’s recruitment sources and how the journal linguistically composes invitations to join the editorial teams. Of note, experience in the field of EDI and understanding of EDI principles alone are insufficient to achieve these goals. Leaders aiming to take on this role should be creative in developing strategies that align with the journal’s aims and resources.
4. ***Establish a mentoring approach***
  There is plenty of evidence showing that members of certain populations are underrepresented in editorial roles. This impedes their ability to receive adequate experience to take on leadership positions. The process of finding editorial board members in all disciplines is challenging as is therefore recruiting editors with diverse backgrounds, gender identities, ethnicities and geographical locations would likely prove to be more challenging. However, a diverse and representative team may be more likely to display increased cultural competency based on their more diverse set of lived experiences. Efforts to recruit a representative team should be in place, and deficiencies in diversity should be explicitly acknowledged as a work in progress. Furthermore, all editorial positions should be time limited as any permanent position of power is prone to propagating disparities.
  Journals can post open calls for reviewer positions rather than solely depending on personal networks to improve the diversity of their reviewer pool. These advertisements should be checked for inclusivity of their wording as well as the locations of their posting. It should be noted that the use of algorithms or artificial intelligence (AI) to identify reviewers, reinforces negative cycle of bias against researchers in low-middle income countries and marginalized populations [54]. Therefore, if AI is used, editors should monitor for potential biases, assess, and mitigate them. In addition, journal editors may encourage authors to recommend reviewers from under-represented backgrounds. Populations carrying the greatest burden of health inequities need a stronger voice in the planning and implementation of their health care and the systems meant to support it, yet for the most part, remain excluded from decision-making processes [55, 56]. Therefore, when inviting reviewers, it may be beneficial to invite reviewers familiar with the article’s content. Knowledge of the author’s name, institution, professional status, or geographical location may result in unconscious bias and abstract the objectivity of the peer review process. To help minimize unconscious bias, journal editors should consider a double anonymized peer review policy where the peer reviewers are not aware of the manuscript's authors and vice versa [37].
  When candidates for journal positions lack experience, establishing a mentoring approach may be a pragmatic approach to preparing them for the role in the future. Senior members of the editorial teams could team up with more junior members and tailor the mentoring according to their needs. Since mentors are highly likely to come from non-underrepresented groups, mentors should receive unconscious bias training or other EDI training as necessary (i.e microaggressions, anti-racism) before engaging in mentorship activities. Given that most editorial positions are voluntary, mentoring activities need to be encouraged and acknowledged to support their work. Mentors could be rewarded by compensating them for their time or establishing internal awards for mentor excellence that may help in promotions and tenures. The uptake of these strategies by several journals may help establish a community of mentors that could be drawn on for mentorship activities. Undergoing training in research integrity may help prepare them for their roles by engaging with their mentees meaningfully and creating a supportive environment. VIRT2UE Train the trainee program is intended for individuals interested in becoming trainers in research integrity. The program focuses on developing behaviours of high moral standards related to the European Code of Conduct for Research Integrity and applying them to specific cases and dilemmas.
5. ***Monitor adherence to equity, diversity and inclusion principles***
  To identify gaps in diversity, meaningful and accurate data collection on the composition of editors, peer reviewers and authors is required. Journal editors need to systematically collect demographic data to accurately assess journal progress and tailor their goals accordingly. A standard list of questions should be presented to the research community where they can voluntarily provide self-identification data such as career stage, gender, race & ethnicity, and geographical location of the journal community [57]. As a first step, journals can use the eight identification categories proposed in the questionnaire distributed by the Employment Equity Act and adjust as appropriate. Alternatively, journals may employ external services, such as TOP factor [58], to monitor journal metrics in implementing EDI principles. Empirical approaches are also needed to determine the effectiveness of the approaches used to improve EDI in academic settings. The UK Research and Innovation summarized interventions, frameworks and outcomes measured to quantitatively monitor changes in EDI interventions. They note the lack of experimental approaches to assess EDI interventions and small sample sizes. Thus, researchers should investigate rigorous approaches to investigate the effectiveness of EDI interventions.
6. ***Publish reports on equity, diversity and inclusion***
  To hold journals accountable for their progress, journals and publishers should make their data on diversity available to the public. Therefore, journals should ensure that they acquire informed consent from participants when collecting their self-identifiable. Their data should be treated with the utmost sensitivity and stored with great care. Although we are not aware of the most appropriate approach to store data, there are ten established rules for storing digital data that journals may apply to safeguard sensitive information [59]. Journals should only present the data in an aggregated form to ensure the confidentiality of participants.

### The role of peer reviewers

Reviewers and journal editors must consider that the author’s first language might not be English. Thus, they must be understanding and try to base their decision on the quality of research rather than the language. If significant language corrections are needed, we suggest directing them to a language service such as SAGE Author Services or Language Editor Services by ElSEVIER and subsequently invite them to resubmit once their manuscript is reviewed. Adjustment may be needed for authors with disabilities or neurodiverse conditions, and peer reviewers should support them accordingly. They may offer them additional feedback, extra time for revisions or arrange a call to discuss feedback.

### The role of researchers

The impact of marginalization on the health of marginalized groups is well established [60]. However, their perspectives are yet to be adequately reflected in evidence bases [55]. The absence of regularly collected data on outcomes and experiences of under-represented populations limits the relevance of available primary evidence informing evidence-based practice. Populations experiencing inequities need a stronger voice in the planning and the implementation of health care services as well as the systems designed to support them. For this reason, they should be involved in decision-making processes [55, 56]. Greater involvement of stakeholders in evidence syntheses can support greater inclusion of social and organizational factors that may influence interventions and review findings [61]. Furthermore, Incorporating EDI in research ensures that pre-conceived beliefs and eco-chamber societies are likely avoided, minimizing confirmation bias and increasing the credibility of research findings [62]. An example of this is The *New England Journal of Medicine* which requires authors to provide the representativeness of the study group in a table as a Supplementary Appendix [63]. They also require authors to appropriately report on the representativeness of the patients included in the study and assess the generalizability of the research findings to populations at risk of experiencing inequities.

Reporting guidelines may improve the reporting of research and should be used by researchers [64–67]. Although guidelines such as the SAGER guidelines have recommended sex-specific analyses to obtain more equitable evidence [68], and several funders have mandated their analyses, such mandates may be insufficient to change reporting practices [69]. Researchers must demonstrate their commitment to improving equity in research by adhering to equity reporting guidelines such as the extensions of the CONSORT (Consolidated Standards of Reporting Trials) [70] and PRISMA (Preferred Reporting Items for Systematic Reviews and Meta-Analyses) [71] more work is needed to assess their impact on reporting.

### The role of universities and academic institutions

Students from under-represented groups face several barriers to success when engaging with academia’s traditional measures and systems of evaluation [4, 30, 72, 73]. A study conducted by Heller and colleagues found that as the GPA score requirements increased in medical schools, in the United States from 2005 to 2009, the diversity of the classes decreased [74]. This suggests that evaluations heavily based on academic metrics often come at the expense of EDI. Thus, establishing a different definition of student academic excellence may help improve EDI in academic institutions.

Several approaches have succeeded in improving diversity among trainees and early-career researchers [75, 76]. However, differential recruitment, retention, and promotion rates across several factors such as age, sex and race are yet to be improved [77–80]. This may be partly due to the narrow focus on citation metrics and publications for the evaluation of these processes [30, 81, 82]. Institutions should award strong mentorship that involve the support of marginalized groups and include tenure or promotion assessments in recruitment. These awards include the National Science Foundation’s Presidential Award for Excellence for Science Math and Engineering Mentoring (PAESMEM), the Australian Museum Eureka Award, and the Nature Research Awards for Mentoring in Science. Expanding the measures of success to include non-academic metrics would enhance the selection of diverse candidates and set the stage for a diverse, new generation of researchers.

Furthermore, academic course coordinators should consider teaching the curriculum from an EDI perspective by diversifying the reading material of courses as well as the research used to compose the learning material [4]. Emphasizing diversity in the educational curriculum fosters the inclusion of diverse students, staff, and relevant topics and better engages underrepresented groups through a curriculum that reflects their lived experiences.

### The role of funding agencies

Several funding agencies, such as NIH [83] and CIHR [84] have acknowledged the importance of equity research. This is integral for improving academia as research funding is indispensable in an academic’s career. Including diversity factors as a scorable criterion may improve research since several studies have shown that diverse teams produce more innovative, creative, and impactful science [81, 85, 86]. Funding agencies could also create grants dedicated to underrepresented scholars to allow more opportunities for them and potentially eliminate the funding disparity in research. Examples include the Mental Health Dissertation Research Grant to Increase Diversity funded by the National Institute of Health [87] and the Louis Stokes Alliances for Minority Participation (LSAMP) funded by the National Science Foundation [88]. Funding agencies could also consider instituting a minimum number of scholars from underrepresented populations as reviewers on funding panels [81]. We acknowledge that this may introduce a “diversity tax” where a burden may be placed on marginalized scholars. However, it is essential to note that the “diversity tax” becomes problematic when the positions and work done are not career enhancing. There needs to be more work on incentivising leadership positions for representatives of marginalized populations in terms of academic value and career progression.

## Conclusions

Journal editors cannot change the culture of academic societies alone since they are constrained by a broader system. Therefore, we advocate for consolidated action for improving EDI by using a systems approach that involves journal publishers, researchers, academic institutions, and funders. We acknowledge the lack of studies that show the effectiveness of interventions aimed at improving EDI. However, we believe that journals adhering to the minimum standards set by RSC and following the guidance suggested in this paper may help journals obtain data that can help monitor their EDI progress. In writing this commentary, we reviewed it for inclusivity and bias-free language. We urge journal editors to develop evaluation plans to measure the effects of EDI interventions in improving the editorial culture using innovative methodological approaches.

## Data Availability

No data was reported in this article.
